# A case of tension pneumoperitoneum with fecal peritonitis due to high‐pressure air insufflation through the anus

**DOI:** 10.1002/ccr3.7344

**Published:** 2023-05-15

**Authors:** Havil Stephen Alexander Bakka, Perumalla Karthik Babu, Lakshmi Venkata Simhachalam Kutikuppala, Tarun Kumar Suvvari, Samrat Babu Koirala

**Affiliations:** ^1^ Department of General Surgery Ramesh Sanghamitra Hospitals Ongole India; ^2^ Department of General Surgery Dr NTR University of Health Sciences Vijayawada India; ^3^ Rangaraya Medical College Kakinada India; ^4^ Nepalese Army Institute of Health Sciences College of Medicine Kathmandu Nepal

**Keywords:** gastrointestinal surgery, peritonitis rectal perforation

## Abstract

**Key Clinical Message:**

The reckless or ridiculous usage of high pressure compressed air could lead to disastrous consequences as demonstrated in this case. Injuries from a barotrauma can vary from a simple mucosal laceration to tension pneumoperitoneum causing abdominal compartment syndrome. Decompression by a wide‐bore needle can be done as depicted in our patient to provide immediate relief.

**Abstract:**

Rectal perforation most commonly occurs due to trauma, but rarely due to a high pressure compressed air passing through the anus as a part of playful joke. Owing to the belief of medico‐legal issues and socio‐psychological circumstances about the ano‐rectal injury, initial approach to the medical facilities might be delayed, causing a delayed presentation and poor prognosis. We report an incident of a young male who presented with tension pneumoperitoneum causing abdominal compartment syndrome with fecal peritonitis due to forceful passing of high‐pressure air through his anus. An initial decompression of the abdomen with a wide‐bore needle was done at the emergency room. An emergency laparotomy with a primary repair of the rectal perforation by two layered sutures was done followed by a loop colostomy, 10 cm proximal to the injury. Colostomy closure was performed after 4 weeks. Post‐operative recovery period was uneventful.

## 1 INTRODUCTION

The use of high pressure compressed air is being readily used in various industrial workplaces for cleaning the dust. The risk of barotrauma (pneumatic injury) resulting from the accidentalor playful use of the equipment has been incident somewhere around the world over time.^1^ Most commonly used tool in these industrial workplaces is the blow gun dust cleaner, which can cause potentially fatal pneumatic injuries if used at different areas of the body like rectum. These pneumatic injuries at the industrial workplaces are rare, but could be devastating.^2^ The air pressure in the dust cleaner is roughly 10 times that of the resting anal pressure, which can readily surpass the resting pressure of anal sphincter, producing vigorous inflation of the colon and rectum. Therefore, the rapid colonic and rectal inflation causes bowel perforation leading to abdominal emergencies like tension pneumoperitoneum. The rectal perforation causing acute abdomen is very rare. Therefore, barotrauma causing rectal perforation becomes furthermore uncommon that needs to be reported for further reference. Due to the rare incidence, industrial employees are unaware of accidental or purposeful usage of high‐pressured air near the perineal region, which can lead to fatal and serious colorectal injuries.^3^ We report a case of bowel perforation with tension pneumoperitoneum causing acute abdominal compartment syndrome with fecal peritonitis and respiratory distress due to barotrauma.

## 2 CASE PRESENTATION

A young man in his 20's presented to the emergency department with severe abdominal pain, abdominal distention, and per rectal bleeding for the past 5 h. On detailed questioning, the patient revealed that a high‐pressure air compressor (7.5 HP) nozzle tip was placed directly into the anus by his co‐workers as a part of a playful joke. The pain score was 8/10 indicating horrible severe pain, which was reported using the Verbal Descriptor Pain Scale.

The patient was conscious, coherent, and dehydrated. On general examination, the patient had dyspnea (O_2_ saturation was 90% on room air), tachycardia (110/min), and blood pressure of 90/60 mm. On systemic examination, CVS—S1, S2 heard, RS—bilateral air entry present, per abdomen revealed gross distention which is tympanic, tender, along with guarding, absent bowel sounds, and liver bed areas was resonant to percussion. Per rectal examination showed no sign of any external injury; however, there was presence of surgical emphysema in the areas surrounding the anus and thigh. Digital rectal examination revealed bleeding from the anus with fecal matter.

**FIGURE 1:**
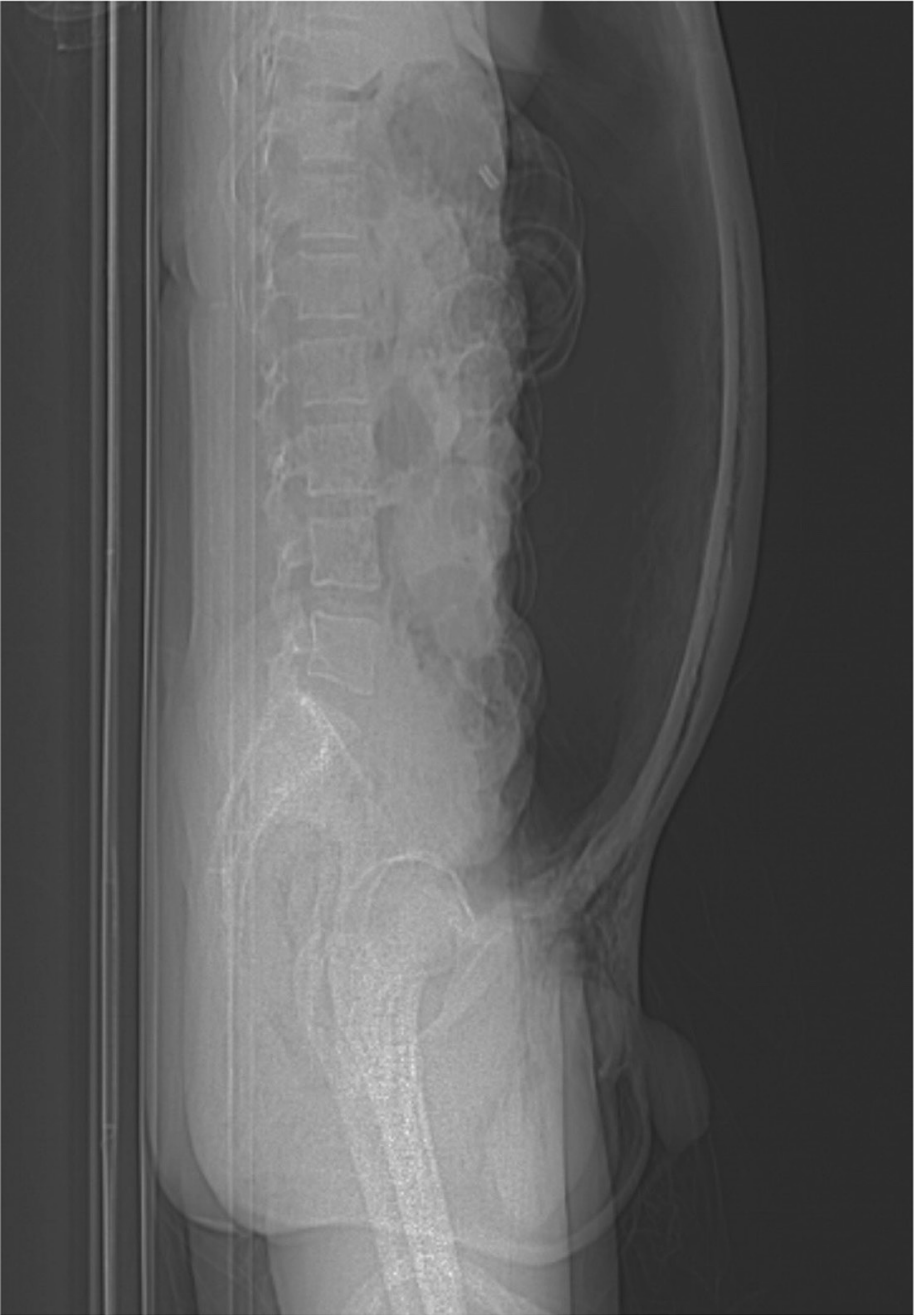
X‐ray erect abdomen showing tension pneumoperitoneum compressing the visceral organs.

**FIGURE 2:**
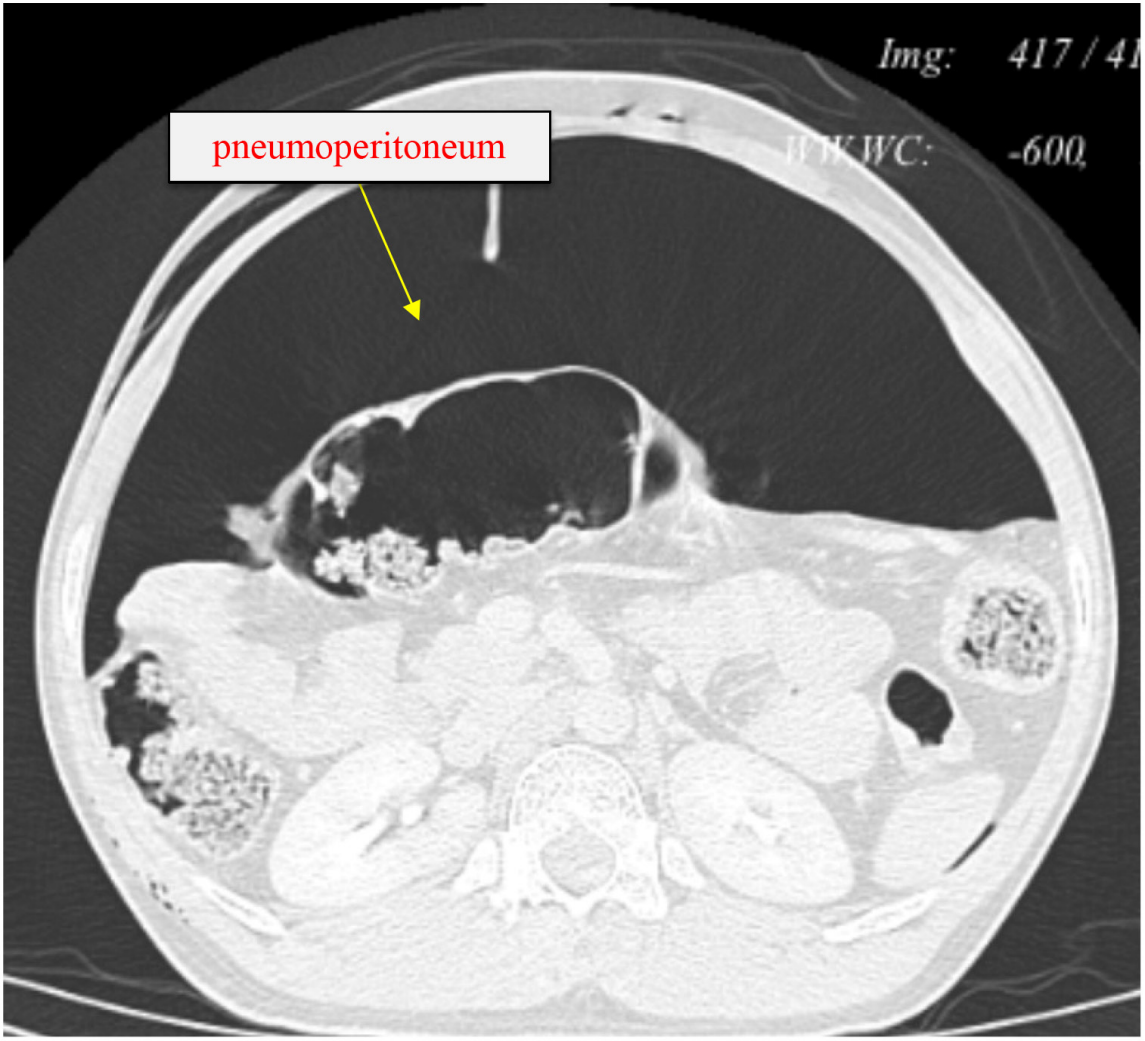
CT abdomen with lung window showing severe pneumoperitoneum predominantly in the anterior aspect causing mass effect over the visceral organs.

Investigations: Routine blood investigations revealed RBC, platelet counts were normal, and hemoglobin was 11.5 g/dL. Arterial blood gas (ABG) revealed metabolic acidosis. An abdominal X‐ray revealed tension pneumoperitoneum compressing the visceral organs (Figure 1). Computed tomography (CT) with and without contrast of the abdomen revealed severe pneumoperitoneum predominantly in the anterior aspect causing mass effect over the visceral organs—the possibility of secondary to bowel perforation (Figure 2). Moderate surgical emphysematous changes in the soft tissues of bilateral perianal regions, bilateral ischiorectal fossa, bilateral scrotal walls, right thigh, and entire abdominal wall.

Treatment: The patient was initially hydrated with intravenous fluids; a nasogastric tube was placed. Urgent abdominal decompression was done with a large wide‐bore needle connected to an underwater seal in the emergency department to decrease respiratory distress as a bedside procedure. Post‐decompression for 15 min distress decreased (saturation increased to 94%); however, pain and distension were still persistent.

**FIGURE 3:**
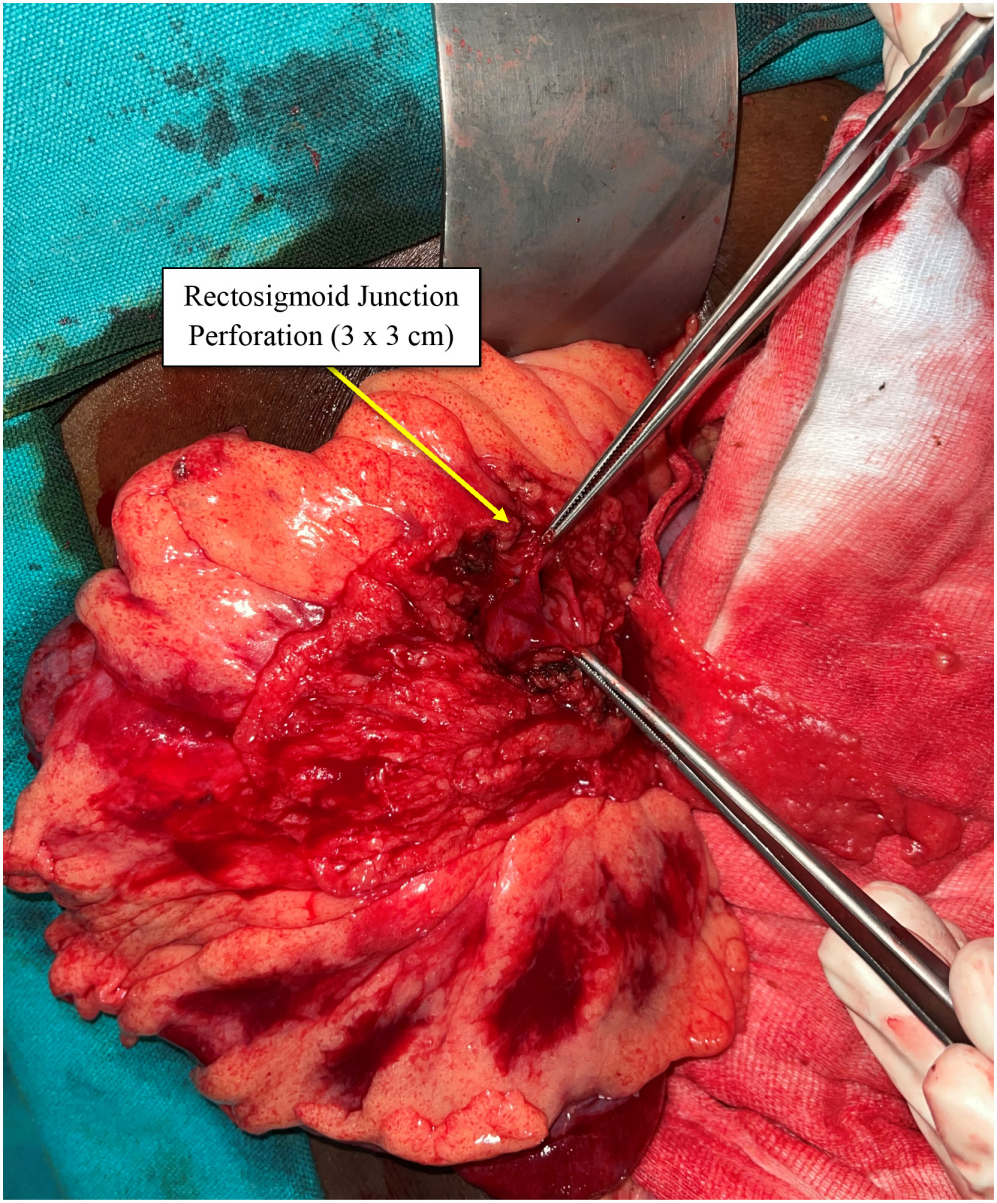
Rectosigmoid junction perforation of size 3 × 3 cm.

**FIGURE 4:**
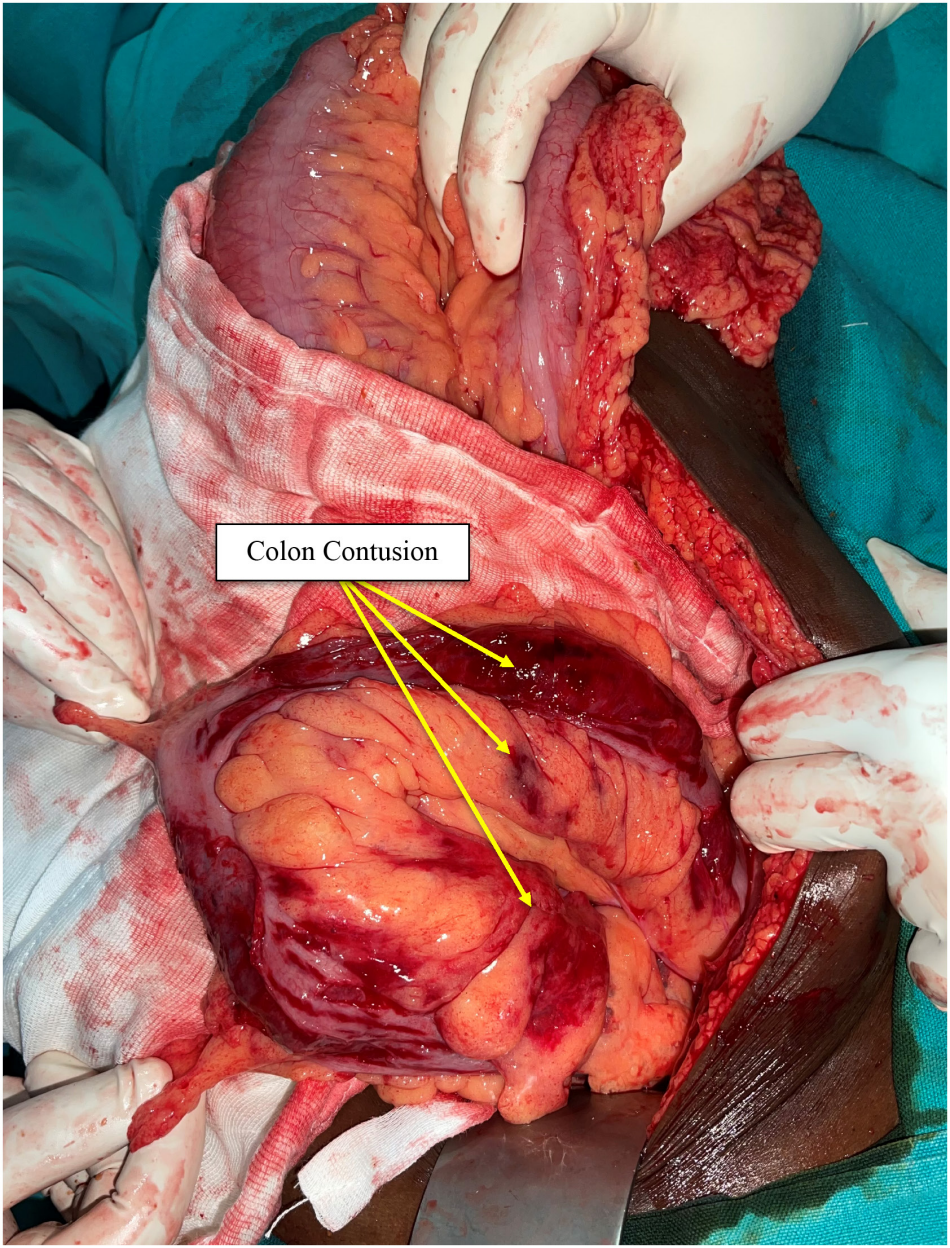
Multiple contusions on the sigmoid colon.

Emergency laparotomy was performed; upon opening the abdomen, the air started to gush out and the peritoneal cavity was filled with fecal fluid. A single rectal perforation of size 3 × 3 cm (Figure 3) was identified with multiple contusions on the sigmoid colon (Figure 4), and the rest of the abdomen was found to be normal. Therefore, the perforation was repaired with a 2–0 Vicryl in a two‐layered fashion by intermittent suture, and a diversion sigmoid loop colostomy was done 10 cm proximal to the site of the injury. A postoperative diagnosis was made as rectal perforation with tension pneumoperitoneum and fecal peritonitis.

Post‐operatively, the patient was extubated on Day 2. Epidural catheterization was performed to manage pain, and total parenteral nutrition (TPN) was given with a kabiven peripheral infusion. Other supportive measures like antibiotics, spirometry were continued along with a unit of packed cell transfusion.

A multidisciplinary team involving a surgeon, critical care specialist, anesthetist, nursing staff, physiotherapist, and nutritionist was involved in the care of the patient.

Outcome and Follow‐Up:

Following discharge, the post‐operative course of the patient was satisfactory and follow‐up was uneventful. Therefore, after sigmoidoscopy, closure of the colostomy site was done in the fourth week. Patient recovered well and is free of all symptoms now.

## 3 DISCUSSION

Barotraumas cause colorectal injuries, which range from minor mucosal tear to massive intestinal perforation, and are relatively infrequent. There have not been many reports of colorectal barotraumas, among which most of them are bowel perforations and some lead to tension pneumoperitoneum.^4^ The majority of these individuals required surgery, and only a handful were treated conservatively. This emphasizes the wide range of clinical manifestations and treatment modalities available in such situations, which necessitates reporting of such injuries and outcomes in order to develop better management regimens.^5^ Rectal trauma affects one to three percent of the patients presenting to emergency departments of tertiary care centers worldwide, with gunshot injuries being the most common cause. However, the rectal perforation as a result of barotrauma to the anus is uncommon. The playful use of high‐pressure compressed air by industrial employees has been linked to severe rectal pneumatic injuries like the one described in this case. Colonic injuries can occur at any location that can range from a simple serosal tear to a full‐thickness perforation. However, rectosigmoid junction and sigmoid colon are the most prevalent sites of injury in such cases due to the relative fixity and angulation, as observed in this patient.^6^ The severity of injuries could be determined by the velocity of airflow, exposure time of the compressed air, resting anal pressure, intraluminal pressure, and bowel distensibility. Rigid proctoscopy along with digital rectal examination must be conducted in cases of suspected rectal damage. Abdominal and chest imaging should be performed in an erect position, by which the enormous amount of air in the peritoneum can be visible. Computed tomography (CT) of the abdomen is the most useful non‐invasive technique for confirming the diagnosis, whereas sigmoidoscopy could be helpful in cases with a negative CT scan.^7^ A huge amount of air enters the peritoneum through the perforation as a result of the high pressure, causing severe abdominal distension and respiratory distress. The use of a wide bore needle for immediate abdominal decompression relieves the distension and respiratory distress. The site of the perforation determines the management of the rectal injuries, whereas the intraperitoneal rectal perforations must be treated surgically.^8^ Resection and anastomosis can be done for managing large defects or injuries. As demonstrated in this case, the reckless or ridiculous usage of high pressure compressed air could lead to disastrous consequences.

## 4 CONCLUSION

Injuries from a barotrauma can vary from a simple mucosal laceration to tension pneumoperitoneum causing abdominal compartment syndrome. Decompression by a wide bore needle can be done as depicted in our patient to provide immediate and temporary relief. However, emergency laparotomy is the definitive management for colonic perforation causing fecal peritonitis with sepsis and shock. A primary repair or resection anastomosis with or without diversion should be performed when required.

## AUTHOR CONTRIBUTIONS

**Havil Stephen Alexander Bakka:** Conceptualization; data curation; investigation; writing – original draft; writing – review and editing. **Perumalla Karthik Babu:** Data curation; investigation; writing – original draft; writing – review and editing. **Lakshmi Venkata Simhachalam Kutikuppala:** Methodology; writing – original draft; writing – review and editing. **Tarun Kumar Suvvari:** Formal analysis; writing – original draft; writing – review and editing. **Samrat Babu Koirala:** Writing – original draft; writing – review and editing.

## FUNDING INFORMATION

None to disclose.

## CONFLICT OF INTEREST STATEMENT

None to disclose.

## CONSENT

Written informed consent was obtained from the patient to publish this report in accordance with the journal's patient consent policy.

## Data Availability

Data sharing is not applicable to this article as no new data were created or analyzed in this study.
